# Helicopter Evacuation Following a Rural Trauma: An Emergency Medicine Simulation Scenario Using Innovative Simulation Technology

**DOI:** 10.7759/cureus.524

**Published:** 2016-03-08

**Authors:** Desmond Whalen, Chris Harty, Mohamed Ravalia, Tia Renouf, Sabrina Alani, Robert Brown, Adam Dubrowski

**Affiliations:** 1 Emergency Medicine, Memorial University of Newfoundland; 2 Faculty of Medicine, Memorial University of Newfoundland; 3 Rural Medicine Education Network, Memorial University of Newfoundland; 4 Offshore Safety and Survival Centre, Marine Institute, Memorial University of Newfoundland; 5 Emergency Medicine, Pediatrics, Memorial University of Newfoundland

**Keywords:** emergency medicine, simulation based medical education, pre-hospital care, rural medicine, remote care

## Abstract

The relevance of simulation as a teaching tool for medical professionals working in rural and remote contexts is apparent when low-frequency, high-risk situations are considered. Simulation training has been shown to enhance learning and improve patient outcomes in urban settings. However, there are few simulation scenarios designed to teach rural trauma management during complex medical transportation. In this technical report, we present a scenario using a medevac helicopter (Replica of Sikorsky S-92 designed by Virtual Marine Technology, St. John's, NL) at a rural community. This case can be used for training primary care physicians who are working in a rural or remote setting, or as an innovative addition to emergency medicine and pre-hospital care training programs.

## Introduction

In rural and remote locations, access to resources for medical management of complex patients is often limited. Patients living in rural areas have higher injury-related deaths than those living in urban areas [1]. Physicians in distant primary care facilities are often required to manage patients themselves or stabilize for transport until they can be transferred to a trauma center for other interventions. In rural medicine, the use of medical transportation is an essential link in the chain of care, and when not available has been shown to increase mortality [2]. Rural physicians must be comfortable with managing complex trauma cases using their available resources. They also need to know how to manage these cases during transport via road, boat, helicopter or fixed wing aircraft.

Trauma management in rural settings poses unique challenges when compared to urban settings. There are usually limited trauma teams in rural and remote communities. Physicians need to have a broad skill set and to be familiar with their limited equipment. Innovation and planning are essential to ensure good patient outcomes and justification for use of medical transport [3].

In Newfoundland and Labrador (NL), 51.7% of the population (265, 772) lives in rural and remote communities according to the 2011 census data [4]. This frequently necessitates the use of medical transportation with aircraft, ambulance or ferry. Simulation-based training in medical transport may be an asset to primary care-based residency programs. Previous studies have shown that up to 12% of patients in emergency medical services (EMS) transport have had misplaced endotracheal tubes when later evaluated [5]. Primary care physicians need to be proficient in the use of limited equipment to perform procedures like intravenous insertion, intubation, and cardioversion [6], often in small, crowded, and moving spaces. 

We describe a scenario based on an actual case from rural NL to teach rural family medicine and emergency residents an approach to rural trauma management and complex medical transportation. 

The learning objectives for this simulation scenario are:

1) To preform an Advaned Trauma Life Support (ATLS) trauma assessment using ABCDE* and projected course

2) Complete initial resuscitation in a rural trauma bay

3) Diagnosis and initial stabilization of head trauma in a rural setting

4) Medical and logistical preparation for transport

5) Management of a complex airway during medical transport

(*A = Airway maintenance with cervical spine protection, B = Breathing and ventilation, C = Circulation with hemorrhage control, D = Disability/Neurologic assessment, E = Exposure and environmental control)

## Technical report

This simulation uses hybrid simulation design, using a combination of high fidelity human patient simulator, confederates, and simulated environments. In this case the patient is the Human Patient Simulator (HPS) (CAE © Healthcare, Quebec, Canada) programed to represent a teenage male with head trauma. The patient is dressed in hockey equipment and skates in order to contextualize the trauma and provide non-verbal cues to the learners. The confederates are Standardized Patients (SPs) from local Standardized Patient Program and they act as an emergency room (ER) nurse and an advanced-care paramedic working with the medevac team. The simulation takes place in a replica of Sikorsky S-92 designed by Virtual Marine Technology, St. John’s, NL.

**Figure 1:**
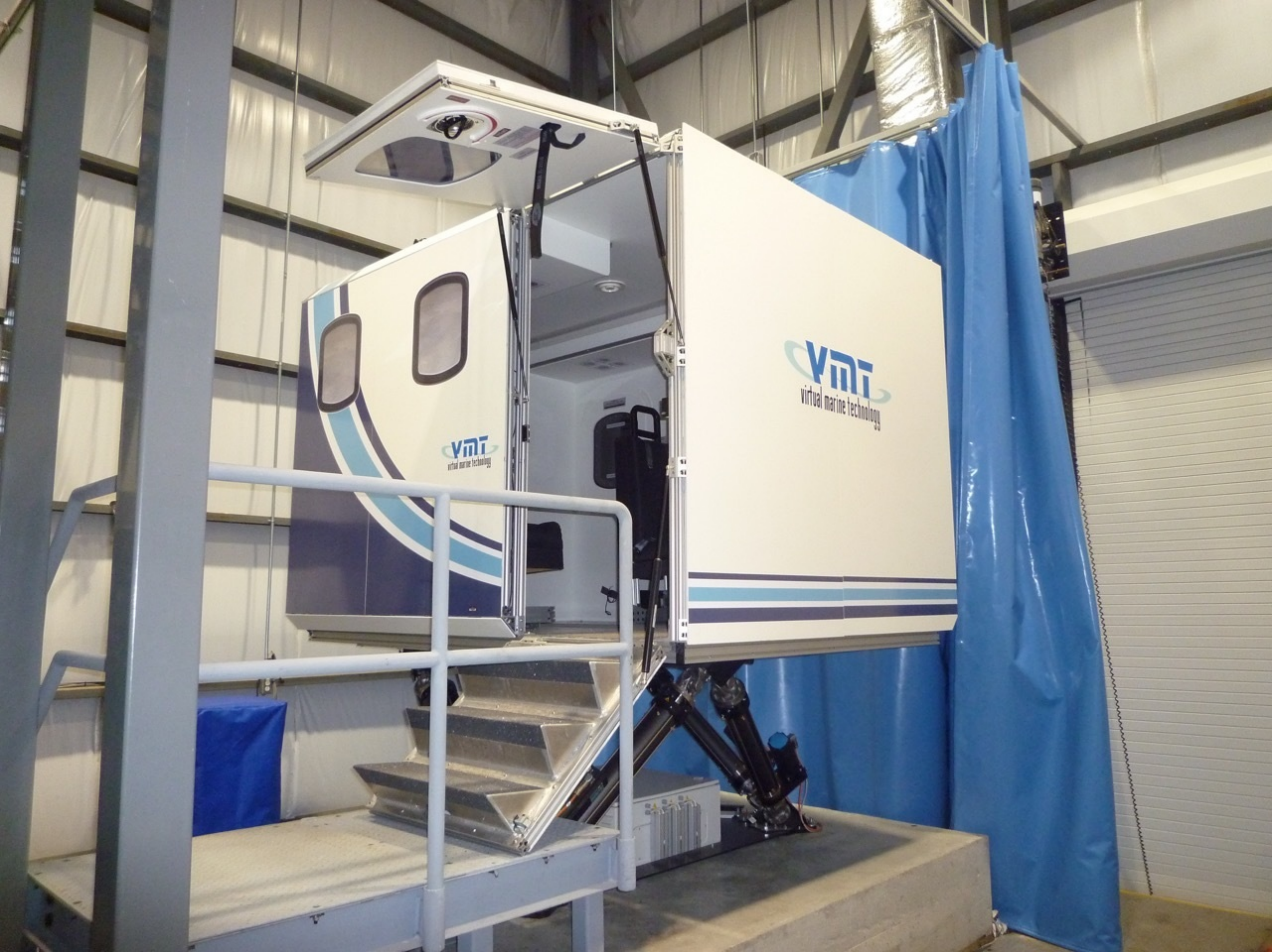
Forward exterior view of marine institute replica of Sikorsky S-92 designed by Virtual Marine Technology

**Figure 2:**
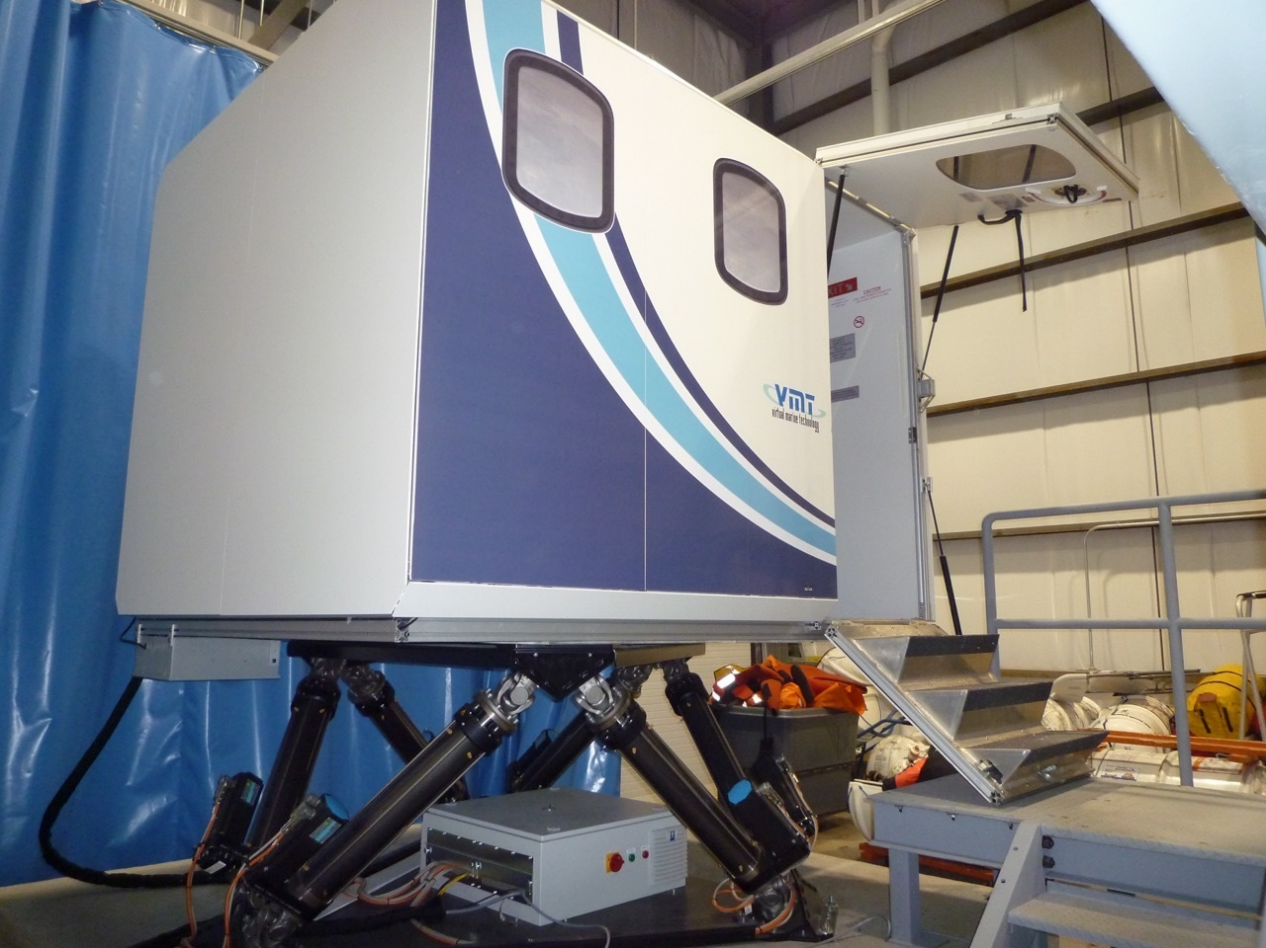
Rear exterior view of marine institute replica of Sikorsky S-92 designed by Virtual Marine Technology

**Figure 3:**
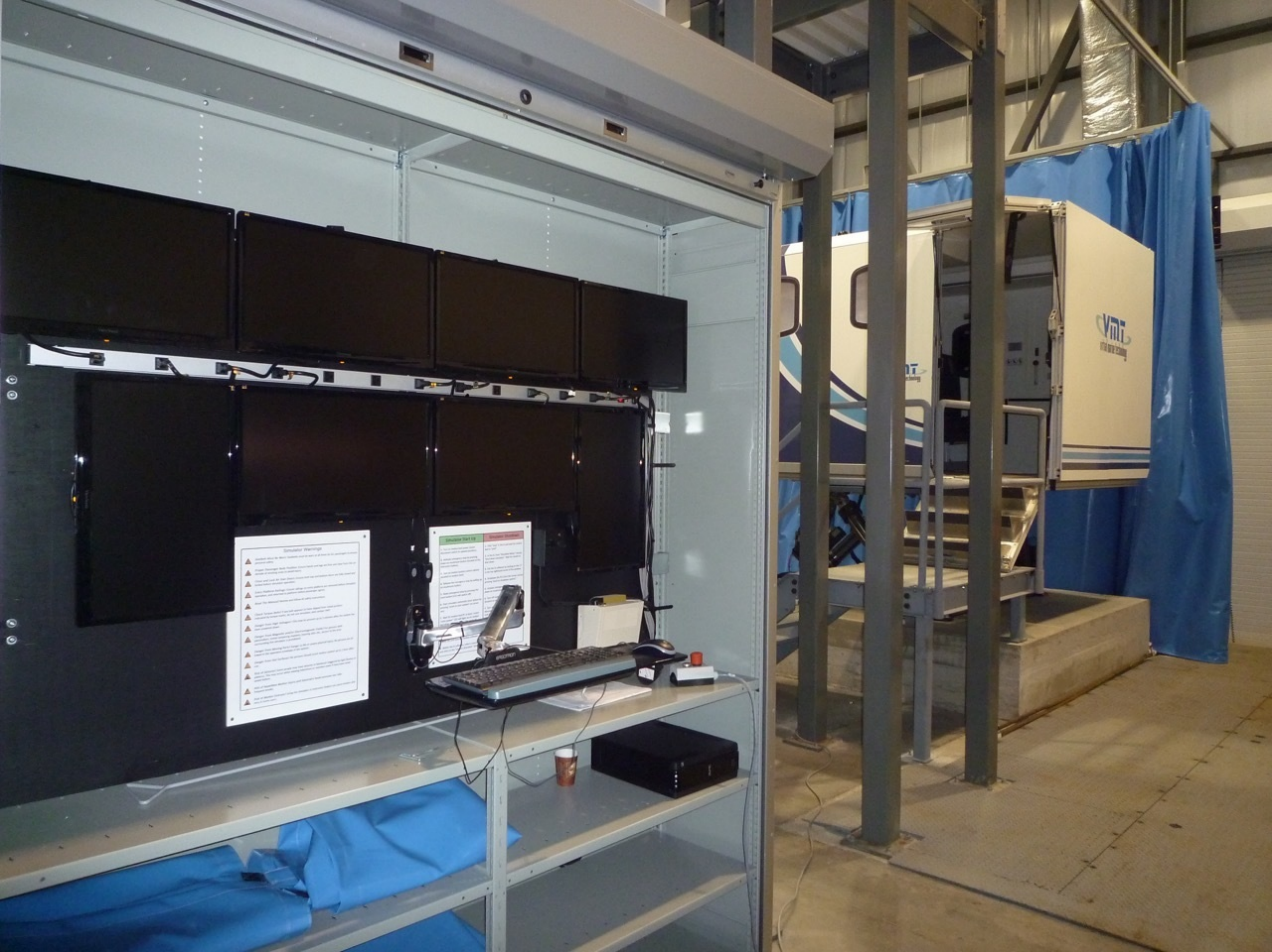
Control panel for marine institute replica of Sikorsky S-92 designed by Virtual Marine Technology

**Figure 4:**
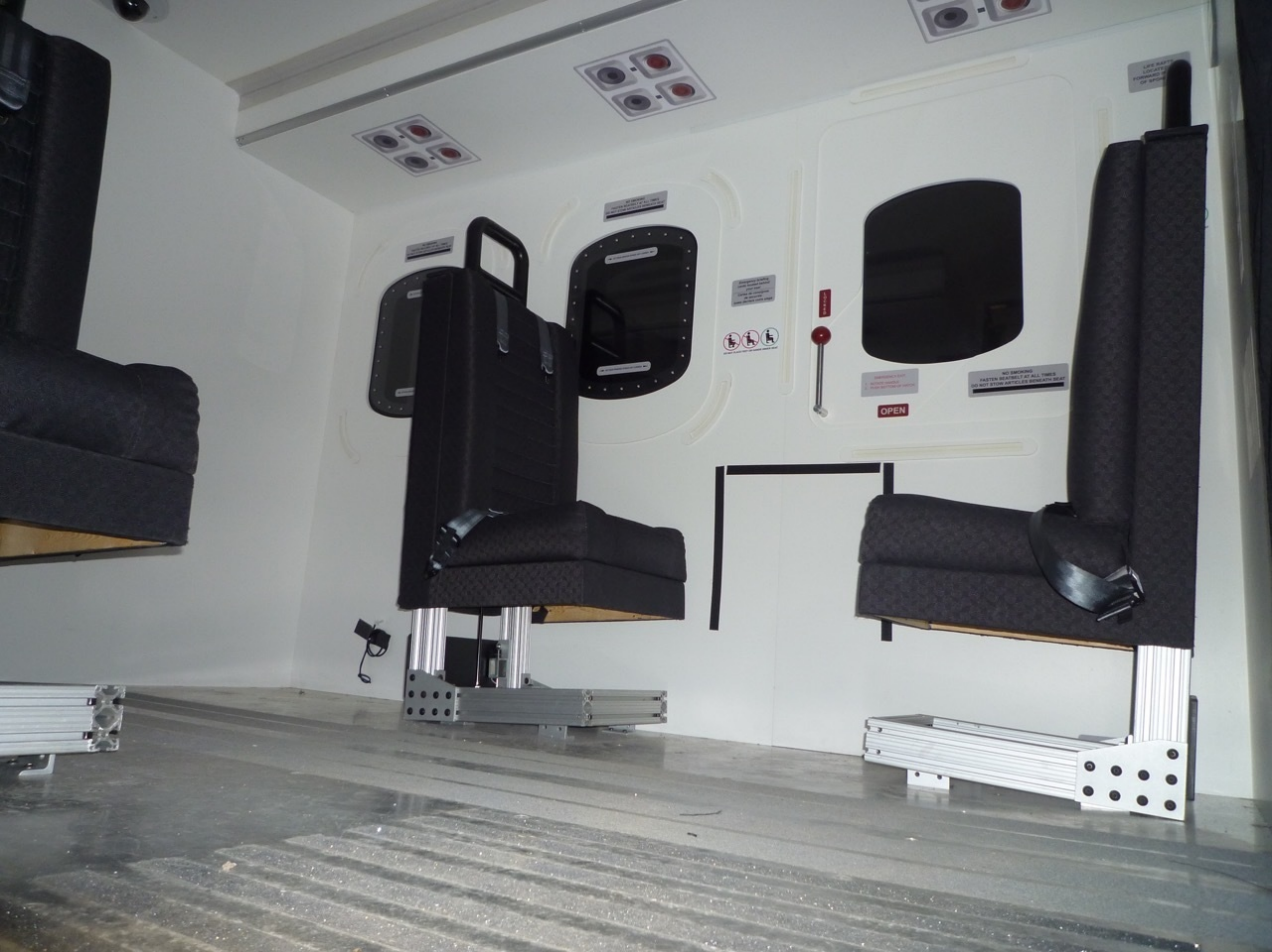
Interior view of marine institute replica of Sikorsky S-92 designed by Virtual Marine Technology

Once medical management in the rural trauma bay is completed, the HPS is transferred to the helicopter simulator, as seen in Figures 1-4, for complex airway management in a moving medevac. The helicopter is pre-programmed to have a 1-minute take off phase, 10-minute flight path with turbulence, helicopter noises, low light, snowy conditions, and a 1-minute landing phase. Additionally, the simulator has a full 6 degree of freedom motion making the flight path realistic. A technician is present at all times to simulate landing the aircraft if requested by the health care team inside the helicopter. The workspace inside the simulator is 2.3 m x 2 m and it contains four chairs, a stretcher with the HPS simulator, an oxygen tank, and two learners. This scenario can also be tailored to other situations where a helicopter simulator is not available. For example, the back of a moving ambulance could be a suitable substitute. In lower-fidelity situations, it is possible to use a small area with audible aircraft sounds designed to act as the tight space found in a medevac helicopter.

**Figure 5:**
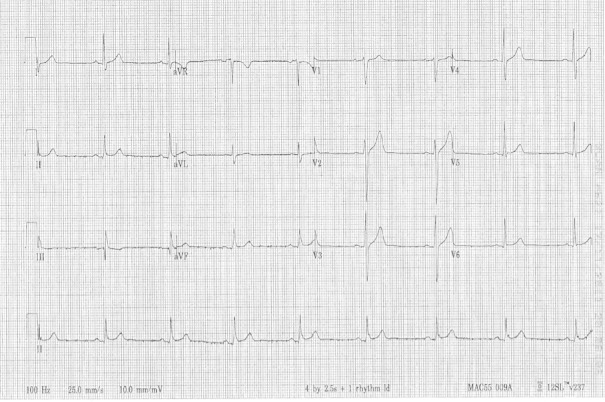
Sinus bradycardia EKG to be provided to learner if requested during scenario

Technical staff program the computer and control the simulation when they are given the stepwise scenario (Table 1). Two confederates are recruited to act as an ER nurse and an advanced-care paramedic working with the medevac team. Alternatively, two family medicine or emergency medicine trainees could learn from the exercise by acting as these confederates. Figure 5 and Table 2 depict additional information to be provided if requested by the learner.

**Table 1:** Scenario template used to program mannequin by technical staff for simulation exercise

Pre-Scenario		
You are an emergency room physician in a remote community. A 15-year-old male is brought into the trauma room by his mother. The teenager was playing hockey on a local pond when he fell and hit the back of his head. The mother informs you that he had a period of unconsciousness but then became responsive again. Since arriving in the trauma bay, his level of consciousness has been waxing and waning. There is a tertiary care trauma center 5 hours away by ambulance and 90 minutes by helicopter medevac. The ambulance is immediately available. The medevac helicopter will take 60 minutes to arrive.		
Begin Scenario – Learner enters the trauma room		
Objective 1: Trauma Assessment and Projected Course		
*Scenario Details*	*Vital Signs/Physical Findings*	*Appropriate Learner Actions*
Mother: “My son fell on the ice! He was unconscious! You need to help!” Mom is distressed and visibly worried.	Vital Signs: BP145/70 / HR 40 / T (Rectal) 35.5°C / RR 22 / SpO2 92% RA. Weight = 52kg	Order: Cardiac and O2 Monitor; Labs (CBC, Electrolytes, BUN, Glucose, INR, Blood type and Screen); 2 Large bore IVs; Preoxygenate; C-spine collar; Broselow tape
	Physical Findings: Patient opens eyes in response to voice (GCS Eyes 3); Patient appears confused and disoriented (GCS Verbal 4); Patient withdraws from painful stimuli (GCS Motor 4); Brisk symmetrical reflexes	Learner Verbalizes: A – Airway is protected with C-spine control; B – Breathing is distressed and tachypnic; C – Circulation is adequate; D –Recognize GCS levels and state that GCS is 11; E – Boggy hematoma on occiput
Mother (yelling): “He seems to be getting worse. Doctor, you need to help.”	Vital Signs: BP145/65 / HR 40 / T (Rectal) 35.5°C / RR 22 / SpO2 92% RA	Order: EKG (See Figure 5); Warm blankets
	Physical Findings: Patient will only open eyes to painful stimuli; (GCS Eyes 2); Patient is making incomprehensible sounds (GCS Verbal 2) Patient has abnormal flexion to painful stimuli (GCS Motor 3)	Lerner Verbalizes/Actions: Recognize decreased GCS and state GCS is 7; Maintain inline C-spine immobilization; Prepare for rapid sequence intubation
Objective 2: Resuscitation		
*Scenario Details*	*Vital Signs/Physical Findings*	*Appropriate Learner Actions*
Intubation with appropriate agents	Vital Signs: BP145/65 / HR 40 / T (Rectal) 35.5°C / / RR 12 / SpO2 98% Bag	Rapid sequence intubation with appropriate agents
If no intubation	Vital Signs: BP145/60 / HR 40 / T (Rectal) 35.5°C / RR 8 / SpO2 82% RA	Initiate rapid sequence intubation with appropriate agents
Warm blankets ordered	Vital Signs: BP145/65 / HR 40 / T (Rectal) 36.5°C / RR 10 / SpO2 98% Bag	
If no warm blankets used	Vital Signs: BP145/65 / HR 40 / T (Rectal) 35.0°C / RR 10 / SpO2 98% Bag	Order warm blankets
Objective 3: Neurological Status and Management		
*Scenario Details*	*Vital Signs/Physical Findings*	*Appropriate Learner Actions*
Reassess vital signs	Vital Signs: BP145/65 / HR 40 / T (Rectal) 36.5°C / RR 12 / SpO2 98% Bag	Verbalize vital signs
Reassess neurological status	Physical Findings: Patient will only open eyes to painful stimuli (GCS Eyes 2); Patient is non-verbal (GCS Verbal 1); Patient extends to painful stimuli in a decerebrate posture (GCS Motor 2). The patient has a dilated left pupil with sluggish reaction to light	Recognize decreased GCS of 5 and combine clinical findings of bradycardia, tachypnea, and hypertension with wide pulse pressure as Cushing’s triad of vital signs with neurological signs of increased cranial pressure indicative of intracerebral hematoma
		Order: Medevac helicopter; Portable chest X-ray (CXR) to assess ET tube placement
		Order: Extra RSI equipment; Extra IV equipment; Portable vitals monitor; Bougie
Check results of ordered tests	Labs: Normal	Consult neurosurgery via telephone
	Type and Screen:TA+	
	EKG: Sinus bradycardia	
ER Nurse: “Neurosurgery advises that patient is critical and needs to be medically transported to tertiary care within 3 hours.”	Vital Signs: BP145/70 / HR 40 / T (Rectal) 36.5°C / RR 12 / SpO2 98% Bag (Patient is intubated)	Order: Status of medevac helicopter. Call in extra staff to accompany patient to tertiary care
Objective 4: Management and Preparation for Transport		
*Scenario Details*	*Vital Signs/Physical Findings*	*Appropriate Learner Actions*
ER Nurse: “Neurosurgery suggests Mannitol 0.5g/Kg IV, 30° elevation of bed, and hyperventilate 30-35 mmHG CO2 as measured by ABG." (if institution has capability) See debriefing connection to objective 4	Vital Signs: BP 145/70 / HR 50 / T (Rectal) 36.5°C / RR 12 / SpO2 98% Bag	Initiate medical management of ICP as indicated by neurosurgery
ER Nurse: “Medevac helicopter has arrived and is awaiting us in the hospital parking lot.”	Vital Signs: BP 130/70 / HR 50 / T (Rectal) 36.5°C / RR 25 / SpO2 98% Bag	Move to medevac helicopter environment
Scenario is moved to helicopter safety simulator in Foxtrap, NL with CAE Human Patient Simulator as patient. Endotracheal tube has already been inserted while in the trauma bay and portable vitals monitor is connected to the patient. Aircraft sounds are easily audible.		
Objective 5: Transportation Management and Difficult Intubation		
*Scenario Details*	*Vital Signs/Physical Findings*	*Appropriate Learner Actions*
Re-check vitals	Vital Signs: BP 140/70 / HR 60/ T (Rectal) 36.5°C / RR 25 / SpO2 95% Bag	Learner Verbalizes: Patient is stable
	Physical Findings: Patient shows tachycardia with pain	Order: Estimated time of arrival to tertiary care hospital from pilot (Answer: 30 minutes); Sedative and analgesia
Medevac Paramedic: “O2 Sats are dropping!” ET tube is removed from patient by technical staff to simulate ET tube slipping out See debriefing connection to objective 5	Vital Signs: BP 140/70 / HR 70/ T36.5°C / RR 10 / SpO2 80% RA	Order: Immediate landing of medevac helicopter; Bag-valve mask respirations by paramedic
		Prepare for re-intubation with bougie
Helicopter lands on stable ground	Vital Signs: BP 140/70 / HR 70/ T36.5°C / RR 10 / SpO2 70% RA	Begin endotracheal tube reinsertion with bougie
Endotracheal tube reinserted with bougie	Vital Signs: BP 140/70 / HR 60/ T36.5°C / RR 10 / SpO2 97% Bag	Instruct pilot to proceed to tertiary care hospital
Rapid sequence intubation not completed	N/A	End Scenario
Medevac helicopter arrives at tertiary care trauma center		
Patient is consulted to neurosurgery and tertiary care trauma team		
End Scenario		

**Table 2:** Laboratory values to be provided to learner if requested during scenario

CBC		
RBC	3.9 x 10^6^ μL	N
HgB	10.5 g/dL	N
HCT	35%	N
MCV	80fL	N
MCHC	32%	N
Reticulocyte Count	--	N
INR	--	N
PTT	35 sec	N
Blood Type and Screen	A+	N
Electrolytes		
Na	135 mmol/L	N
Cl	105 mmol/L	N
K	3.7mmol/L	N
Mg	1.8 mmol/L	N
Ca	2.4 mmol/L	N
PO4	1.8 mmol/L	N
Chemistry		
BUN	2 mmol/L	N
Glucose	2.2 mmol/L	N
Cr	28.1 μmol/L	N

Pre-briefing

Learners are introduced to the “fictional contract” prior to the beginning of the scenario. This means they acknowledge the fictional component of the case, but will behave as though it is real. This ensures a safe environment where learners can make mistakes. We identify the lead for the scenario, review each participant’s role, and identify the technical staff to the learner. Any limitations to the scenario, including technical limitations of the mannequins and the helicopter are reviewed. Finally, we inform learners whether the assessment is formative or summative, and that the results will be used either for continuing education and skills improvement, or for exam purposes. A checklist is provided in the appendix that may aid in formative or summative assessment of learners.

Case

The learners are informed that a 15-year-old male (a CAE© Human Patient Simulator) is brought to the rural emergency room after a fall on the ice. The patient is unresponsive. The mother informs the learners that the boy struck the back of his head and became unresponsive after the injury. He regained consciousness for five minutes and his consciousness has been waxing and waning since entering the trauma bay.

Another learner, or trained standardized patient, acts as the ER nurse. The scenario is set in a rural emergency room with access to a standard resuscitation cart. The medications and equipment for rapid sequence intubation are readily available. An X-ray is available, but there is no CT scanner. The nearest trauma center is five hours away by road and 90 minutes away by helicopter. An ambulance is readily available. A helicopter medevac will take 30 minutes to arrange. Given the severity of the injury, the learner should recognize the immediate need for helicopter evacuation. However, this is not a learning objective and the learner will be instructed to use a helicopter medevac. Post scenario didactics can include a discussion of other transportation types and their appropriateness to this case.

After the patient is intubated, the scenario is moved to the back of a medevac helicopter. A second confederate takes on the role of a paramedic. Mid-flight, the endotracheal tube becomes dislodged. The pilots land the helicopter so the learner can re-intubate the patient in a semi-controlled setting. The learner has access to rapid sequence intubation equipment.

Two trained emergency room physicians act as instructors. One is designated as the exercise lead and the other assesses the learner and takes notes. The lead ensures that the technical staff follow the template, verifies clinical accuracy, troubleshoots and provides the learners with supplemental materials as requested. The other emergency room physician uses a pre-developed guideline to record performance and assess technical skills. This report is used for formative assessment and debriefing following the scenario. In examination situations, the developed checklist could be used for summative assessment purposes.

The instructors rehearse the scenario ahead of time to ensure limitations are identified and technical issues are resolved.

A video (Video 1) showing a complete run-through of this scenario is included as an Appendix. It is intended to provide a step-by-step approach to the case.

Debriefing

Following the scenario, each trainee participates in a debriefing exercise, an important part of simulation teaching [7]. They are encouraged to openly express their feelings about the case, their perceived limitations and whether the overall experience was positive or negative. An equal number of learners to instructors participate in the debriefing exercise to foster openness. An internal process of debriefing using a combination of frame discovery [8] and the 3D model of debriefing [9] are used. The learners first describe their thought process during the exercise. The instructors then offer neutral suggestions for different approaches in the context of the learners’ thought processes. An alternate method of debriefing—Promoting Excellence and Reflective Learning in Simulation (the PEARLS framework)—is also an option, depending on the preference of the simulation group [10].

In the debriefing, the instructors ask the learners specific questions about other possibilities during the exercise. While our script follows a definite plan, the case can take many different directions. The following questions can be used as links to reinforce specific objectives in the scenario that are not addressed during simulation exercise.

Objective 4 Prompt:

During the scenario, the neurosurgical consultant advises that you intubate, elevate the head of the bed, and hyperventilate as management for a suspected increased intracranial pressure (ICP). In a rural and remote setting what other intervention could be considered if an epidural bleed is suspected? (Answer: Burr Hole). It is important to address with the learner that this is only performed if one pupil is dilated, and the burr hole is to be placed on the side of the dilated pupil.

Objective 5 Prompt:

During the scenario, the medevac paramedic instructs that the SpO2 is decreasing during flight. The reason for this is found to be a dislodged endotracheal tube. What other factors could lead to a decreased SpO2 in this situation? (Answer: the need for increased FiO2, pneumothorax, inadequate bagging, kink in ET tube).

Post-scenario didactics

A brief didactic component after the scenario addresses learning objectives and key components of the ATLS protocol, as well as clinical pearls of transportation medicine. This provides the opportunity for immediate identification of knowledge gaps noted during the scenario, and the chance to consolidate pertinent clinical information. Key information for the didactic session is presented in Table 3 and comes from research on airway management [11], rapid sequence induction [12], and management of traumatic brain injury [13].

**Table 3:** Suggested discussion points to be used for post-scenario didactic teaching session

Scenario Component	Teaching Points
Airway Management	Common airway problems encountered in head traumas include a loss of respiratory drive or altered level of consciousness requiring intubation and ventilation, as well as airway secretions and blood that obscures the airway. C-spine injuries are common in head injury, so ensuring proper C-spine immobility through airway assessment is crucial. In this simulation, decreased level of consciousness and increased cranial pressure make intubation a high priority. Following rapid sequence intubation, endotracheal tube placement must be confirmed using methods such as visualization through the vocal cords, auscultation, and end tidal CO2 detectors. A chest X-ray can rule out an ET tube placement in the right bronchus. Depending on the available equipment, clinical methods (ie visualization and auscultation) may have to suffice until transfer to a tertiary site is complete.
Rapid Sequence Intubation	In rapid sequence intubation (RSI), preoxygenation maximizes the patient’s SPO2 levels. Induction agents (propofol, ketamine or etomidate) and neuromuscular blocking agents (succinylcholine or rocuronium) are then used. In this case, given the head injury, ketamine should be avoided as it poses the risk for hypertensive episodes. Rapid administration of these agents is performed intravenously to facilitate rapid endotracheal tube placement. Patient positioning techniques such as laryngeal manipulation can facilitate cord visualization. Following rapid sequence intubation, endotracheal tube placement must be confirmed using methods such as visualization through the vocal cords, auscultation (the gold standard), and end tidal CO2 detectors. A chest X-ray can rule out an ET tube placement in the right bronchus. Depending on the center and limited equipment, clinical methods (ie visualization and auscultation) may have to suffice until transfer to a tertiary site is complete.
Neurological Assessment	Neurological assessment shows a decreased level of consciousness and dilated pupils. Cushing’s triad vitals are observed: bradycardia (see Figure 1), tachypnea, and hypertension with wide pulse pressure. This suggests increased intracranial pressure (ICP) and a potential intracerebral bleed. Prompt neurosurgical assessment and intervention at a tertiary hospital will be necessary.
Environmental Assessment	Exposure of the entire patient to assess additional injuries is the next key component of the exam. Hypothermia in the setting of acute epidural bleed is dangerous and needs to be prevented and treated. Warmed IV solution and blankets can be used to warm the patient.
Air Transport	A chest X-ray prior to air transportation rules out pneumothorax, which can become a medical emergency at altitude. Air transport is a unique challenge because oxygen levels decrease at higher altitudes. In healthy subjects, an 8000 ft increase in altitude decreases the oxygen saturation by 4%. This mandates continuous assessment and management of a patient’s oxygenation status throughout the transport. During transportation, adequate sedation must be achieved through bolus dosing or a continuous IV infusion of induction agents. The level of sedation can be assessed by response to a painful stimulus. A tachycardic response indicates that a deeper level of sedation and analgesia is needed.

## Discussion

The scenario is designed to teach principles of trauma and head injury management in a rural setting where resources are limited and immediate access to specialists is lacking. The scenario uses only one confederate in the ER and one confederate in the helicopter. This illustrates that few personnel may be available in rural and remote areas.​

Trauma scenarios are frequently encountered by rural physicians [1]. In rural and remote areas with few resources, rural physicians must use sound clinical judgment to make decisions without the help of colleagues or sophisticated medical equipment. Rural simulation scenarios can prepare urban students and practicing physicians to learn and work outside tertiary care centers. 

Simulation-based medical education improves physicians’ knowledge and skills [14]. In this trauma simulation, we place a high-fidelity mannequin in a simulated helicopter environment in order to teach principles of trauma management and rural transport.

In rural and remote communities, trauma cases are often transported to a tertiary center. The physicians doing these transports must be aware of the associated potential risks and challenges. Endotracheal tube dislodgement is an airway emergency. Prompt re-intubation must be accomplished as soon as possible in a stable environment, with adequate evaluation of tube placement [5]. In the event that a stable environment could not be achieved (ie. the helicopter is unable to land) the physician must be able to re-intubate in a small moving and noisy environment. 

## Conclusions

We simulate a critical rural trauma case that requires helicopter transport to a tertiary care center. Such cases are uncommon in rural and remote practice, but necessary for effective patient management. Simulation-based medical education is an effective way to teach the skills that are necessary to manage these cases.

## Appendices

The researchers have developed a video showing a complete run-through of this scenario. This video is intended to provide a step-by-step approach to the case. 

The researchers have developed a checklist to be used for formative or summative assessment of learners. 

**Table 4:** Checklist for formative or summative assessment of learners

Scenario Assessment Checklist	Completed	
	Yes	No
Objective 1 (Trauma Assessment)		
Ask for vital signs		
Order Cardiac and O2 Monitor		
Order CBC, Electrolytes, BUN, Glucose, INR, T&S		
Order 2 Large Bore IVs		
Initiate oxygen therapy		
Verbalize ABCDE findings		
Maintain C-spine inline immobilization		
Order EKG		
Order warm blankets		
Reassess ABC on an ongoing basis		
Objective 2 (Resuscitation)		
Recognize projected course		
Rapid Sequence Intubation with appropriate agents		
Objective 3 (Neurological Status)		
Reassess vital signs		
Reassess neurological status (GCS)		
Review lab results		
Recognize need and perform Neurosurgery Consult		
Receive orders from Neurosurgery for management		
Order Medevac Helicopter		
Objective 4 (Preparation for Transport)		
Administer Mannitol		
Elevate head of bed		
Initiate hyperventilation		
Ensure all equipment is ready for transport		
Objective 5 (Difficult Intubation)		
Reassess vital signs		
Recognize the need for additional analgesia (HR increase with pain)		
Recognize airway instability (dislodged ET tube)		
Order immediate landing of helicopter		
Appropriate re-intubation		
Reassess vital signs and ABCs before takeoff		
Order transport to tertiary care center		
Appropriate handover to tertiary care		
